# “It Is A Carrot-Stick Model”: A Qualitative Study of Rural-Serving Clinician and Rural-Residing Veteran Perceptions of Requirements to Quit Smoking prior to Elective Surgery

**DOI:** 10.1155/2023/3399001

**Published:** 2023-11-30

**Authors:** Sara E. Golden, Allison Young, Christina J. Sun, Marissa Song Mayeda, David A. Katz, Mark W. Vander Weg, Kenneth R. Gundle, Steffani R. Bailey

**Affiliations:** ^1^Center to Improve Veteran Involvement in Care, VA Portland Health Care System, Portland, OR, USA; ^2^Department of Pulmonary, Allergy, and Critical Care Medicine, Oregon Health & Science University (OHSU), Portland, OR, USA; ^3^College of Nursing, University of Colorado Anschutz Medical Campus, Aurora, CO, USA; ^4^School of Medicine, OHSU, Portland, OR, USA; ^5^Department of Internal Medicine, University of Iowa Health Care, Iowa City, IA, USA; ^6^Center for Access & Delivery Research and Evaluation (CADRE), Iowa City VA Health Care System, Iowa City, IA, USA; ^7^Department of Community and Behavioral Health, University of Iowa, Iowa City, IA, USA; ^8^Department of Orthopaedics and Rehabilitation, OHSU, Portland, OR, USA; ^9^Operative Care Division, VA Portland Health Care System, Portland, OR, USA; ^10^Department of Family Medicine, OHSU, Portland, OR, USA

## Abstract

**Introduction:**

Some medical centers and surgeons require patients to stop smoking cigarettes prior to elective orthopaedic surgeries in an effort to decrease surgical complications. Given higher rates of smoking among rural individuals, rural patients may be disproportionately impacted by these requirements. We assessed the perceptions and experiences of rural-residing Veterans and clinicians related to this requirement.

**Methods:**

We conducted qualitative semistructured one-on-one interviews of 26 rural-residing veterans, 10 VA orthopaedic surgery staff (from two Veterans Integrated Services Networks), 24 PCPs who serve rural veterans (14 VA; 10 non-VA), and 4 VA pharmacists. Using the knowledge, attitudes, and behavior framework, we performed conventional content analysis.

**Results:**

We found three primary themes across respondents: (1) knowledge of and the evidence base for the requirement varied widely; (2) strong personal attitudes toward the requirement; and (3) implementation and possible implications of this requirement. All surgery staff reported knowledge of requirements at their institution. VA PCPs reported knowledge of requirements but typically could not recall specifics. Most patients were unaware. The majority of respondents felt this requirement could increase motivation to quit smoking. Some PCPs felt a more thorough explanation of smoking-related complications would result in increased quit attempts. About half of all patients reported belief that the requirement was reasonable regardless of initial awareness. Respondents expressed little concern that the requirement might increase rural-urban disparities. Most PCPs and patients felt that there should be exceptions for allowing surgery, while surgical staff disagreed. *Discussion*. Most respondents thought elective surgery was a good motivator to quit smoking; but patients, PCPs, and surgical staff differed on whether there should be exceptions to the requirement that patients quit preoperatively. Future efforts to augment perioperative smoking cessation may benefit from improving coordination across services and educating patients more about the benefits of quitting.

## 1. Introduction

Cigarette smoking is associated with the development of complications after surgical procedures, including increased postoperative wound healing time, wound infections, and cardiovascular complications [1, 2]. The risk of adverse events, as well as payment or performance models that incentivize reductions in surgical complications, have led surgeons and hospitals, including some Veterans Health Administration medical centers, to implement policies that require patients to quit smoking cigarettes prior to elective orthopaedic surgeries. The impact of this smoking cessation requirement is particularly salient for veterans living in rural areas, who have higher rates of smoking than those living in urban and suburban areas [3]. Rural-residing patients also have limited access to specialty care compared to their urban counterparts [4].

Given the incentive of a desired surgery, this preoperative period could be an opportune time to assist patients to quit smoking; however, studies have shown that perioperative smoking cessation assistance is not consistently provided [5, 6]. In previous work, we found that clinician-level knowledge and system-level implementation of requirements to quit smoking vary widely [7]. While some clinicians feel that primary care is the optimal location to provide preoperative smoking cessation services, primary care and surgical clinicians frequently lack the knowledge and ability to manage a patient's cessation journey before and after elective surgery [8]. This could be due to lack of care coordination or communication, fear of harming the patient-clinician relationship, insufficient training on intervention techniques, or limited knowledge related to requirements (e.g., use of medications and length of abstinence). Further, primary care clinicians and surgery staff may have little time or knowledge about the perioperative effects of smoking on surgeries, which may in part be why patients continue to smoke [5, 9, 10].

We know of no studies that have examined perceptions of both patients and clinicians regarding preoperative smoking cessation requirements. Engaging with rural-residing veterans and clinicians to understand their perspectives toward smoking cessation requirements may help to inform intervention strategies to facilitate the delivery of recommended tobacco use treatment services, support perioperative cessation efforts in this underserved population, and to reduce potential adverse consequences related to the requirement. Thus, we evaluated the perceptions and experiences of rural-residing veterans, rural primary care providers (PCPs), and veterans administration (VA) orthopaedic surgery staff and pharmacists related to the requirement for patients to quit smoking prior to elective orthopaedic surgery.

## 2. Methods

We conducted qualitative interviews with rural veterans, VA pharmacists, and orthopaedic surgery staff (including surgeons, APPs, and nurse coordinators on the operative team), and both VA and non-VA primary care providers (PCPs) who serve rural veterans. We conducted a single one-on-one semistructured interview with all participants by phone or online video platform between February 2021 and July 2022 to evaluate knowledge, attitudes, and behaviors associated with preoperative smoking cessation requirements. Interviews ranged from 15 to 39 minutes for patients and 22-42 minutes for clinicians. Ethics approval for this project was given by the joint VA Portland Health Care System (VAPORHCS)/Oregon Health & Science University (FWA00000161; IRB00000471). All participants completed informed consent (VA Portland Health Care System (VAPORHCS)/Oregon Health & Science University IRB#22100).

### 2.1. Sample

We mailed study invitation letters to veterans from rural Oregon identified via the VA Corporate Data Warehouse, a national repository of clinical and administrative data from veterans receiving care through VA. We selected patients identified as currently smoking by either standard EHR entries or cotinine lab values, prior to, or at the time of, a consult for elective orthopaedic surgery at the VAPORHCS. Of note, the VAPORHCS is the referral center for all of Oregon veterans' elective surgeries.

We recruited VA clinicians via email from two Veteran Integrated Service Networks, which represent regionalized care systems, through a combination of key informant and snowball sampling [11], as well as collecting clinician names from contact lists on the VA intranet. We recruited non-VA PCPs through email contact by colleagues at the Oregon Rural Practice-based Research Network, a practice-based research network that conducts research and quality improvement projects with clinics across the state [12]. There were no exclusion criteria for VA orthopaedic surgery staff. The only inclusion criterion for PCPs and pharmacists was that they had to serve rural-residing veterans. Non-VA clinicians were reimbursed $100, and veterans were reimbursed $50 for completion of interviews.

### 2.2. Data Collection and Analysis

We used semistructured interview guides that focused on participants' knowledge and perceptions of the requirement for preoperative smoking cessation as well as reactions to the requirement (Appendix A). We used probing questions with participants to gain more information. One social science investigator (SEG) conducted the majority of the interviews; a trained research assistant performed two. We achieved saturation of the main themes [13, 14]. The interviews were digitally recorded, professionally transcribed verbatim, and verified for accuracy. Participants self-reported demographic and smoking characteristics during the interview. Participants herein are given anonymous identifiers: “IDP” for patients or “ID (non-VA/VA)” for clinicians (note that all surgeons were VA).

We used ATLAS.ti 22 (ATLAS.ti GmbH, Berlin, Germany) to organize and support conventional content analysis of the qualitative data. SB (psychologist/principal investigator) and SEG (social scientist) each read the first three interviews, created preliminary codebooks, and iteratively refined the codebook and coding throughout. The codebook was considered final for clinicians after a review of ten transcripts and for patients after a review of six. We continued to code every other interview together, evaluating any overlapping coding or uncoded text to verify appropriateness. We developed initial and integrative memos throughout to capture thoughts or analytic ideas, in accordance with the knowledge, attitudes, and behavior framework, which aided in the final interpretation of the data in matrix form by identifying patterns and variations in the transcripts. We also utilized an audit trail for tracking of modifications and decisions related to the codebook and qualitative analysis.

We utilized a knowledge, attitudes, and behavior (KAB) framework for our analysis. KAB theory proposes that health knowledge and information provide a foundation for establishing constructive attitudes and beliefs toward disease, which in turn drive changes in patient behavior [15]; it has also been adapted and used to evaluate clinician behavior and adherence to guideline-recommended care [16, 17]. Herein, we only describe knowledge and attitudes, as behavior will be assessed in our next steps after implementation of a subsequent intervention to address smoking cessation for veterans offered elective orthopaedic surgery.

## 3. Results

We interviewed 26 rural-residing veterans, 10 VA orthopaedic surgery staff (from two VISNs), 24 rural PCPs (14 VA; 10 non-VA), and 4 VA pharmacists. Self-reported characteristics can be found in Table 1. We found three main themes across both clinicians and veterans: (1) knowledge of and the evidence base for the requirement varied widely; (2) strong personal attitudes toward the requirement; and (3) implementation and possible implications of the requirement.

1. Knowledge of the requirement and the evidence base for the requirement varied widely across participants.

### 3.1. Clinicians

All surgery staff reported some knowledge of requirements at their VA and/or affiliated academic institution. They also acknowledged that implementation of requirements often varied by institution, VISN, and by surgeon, even within institutions with recommendations for cessation. One surgeon (3 (VA)) noted: “I'm not sure the exact wording of the policy. I know that it's strongly encouraged for patients to stop smoking, but I'm not certain that it was a hard and fast thing… And so I think that there's areas for physician discretion.” Another surgery staff member (6 (VA)) said, “Since 2016 the recommendations become a strong recommendation, guideline toward a patient… indicated for surgery has to have a negative tobacco or nicotine in the serum. And exempt would be very, very rare and then in a very special circumstance.” Two surgery staff specifically noted that the requirement was a standard of care, not a “policy,” since it was not written down or authorized by a wider VA umbrella organization. All surgery staff adopted a requirement for patients to quit, although some allowed exceptions (see theme 3).

PCPs and pharmacists varied in their knowledge of such requirements. Most non-VA PCPs did not know VA's requirements or if one existed. VA PCPs and pharmacists reported knowledge of a requirement, but they could not recall specifics such as if biochemical verification was required or the timeframe for quitting. They reported relying on referral templates within the electronic health record (EHR), describing difficulty in keeping track of different and changing requirements for referrals. There were a handful of questions, mostly from PCPs, about the actual evidence base behind these requirements. For instance, PCP 11 (non-VA) stated, “it would be interesting to look at the statistics… if there's a really good research study already out there that shows the importance of the surgical, postsurgical healing for nonsmokers versus smokers. And whether it really does make a difference.” There were also questions from PCPs and pharmacists about the evidence behind, and requirements related to, the use of nicotine replacement therapies. Surgical staff requirements related to nicotine replacement varied.

### 3.2. Patients

Many patients were unaware of such a requirement until it was either brought up by surgery staff or by the researcher during the interview. Of note, not all patients had undergone surgery (65% had not) at the time of the interview, which may have impacted their knowledge level. Of those who were aware, only a couple knew the timeframe required to be quit prior to surgery. For example, 29P said, “one of the requirements there is they absolutely will not do the surgery until you're six weeks out on nicotine.” Patients did not have many questions, but those who did expressed desire to know why the requirement exists and, like PCPs, if there is evidence behind the requirement. A few also questioned why they (or others) were able to smoke during other surgeries but not for this one. For instance, one patient said: “I understand that it is detrimental to the healing process… But on the other end of that, I've done a little research, and I know that there have been several thousands, if not hundreds of thousands of these surgeries performed on people who smoke, and those people have had successful complete full recoveries” (4P).

(2) Strong personal attitudes toward requirement and its ability to motivate patients to quit

### 3.3. Clinicians

The majority of clinicians felt this requirement could increase motivation to quit smoking. Although a few surgeons thought there were higher priorities than cessation when thinking about eligibility for surgery, they all agreed that the requirement was there to optimize outcomes and was in the best interest of the patient. One surgery staff member (4 (VA)) said, “I think this [requirement] is a way to encourage patients to stop… ‘cause there's a carrot at the end of it. It's a surgery that they need.” Another surgeon (5 (VA)) said, “I was pretty amazed about these number of [surgeons] that didn't truly have set criteria for this. ‘Cuz I feel like it's pretty night and day in the orthopedic literature on how people do.” Surgeon 3 (VA) said what many echoed: “I think it's actually reasonable to have the patient have some skin in the game so to think toward trying to improve their recovery and optimize them for having the best outcomes and the best recovery.”

Most PCPs felt the requirement was reasonable given the evidence. For instance, PCP (23 (VA)) said, “I support it… I can't really see the utility in putting people through large procedures when they are at higher risk because of their smoking… and then they're just gonna continue to do the thing that made the problem in the first place.” A couple of PCPs thought the requirement was unethical since it discriminates along socioeconomic lines. PCP 15 (non-VA) expressed: “I really think about how like smoking hits across sort of certain demographics… smokers tend to be in lower SES strata, and they're often insured on Medicaid, and so that already affects their ability to access some of these resources. And it's just like yet another F-U to them. Sorry, like you had to wait six months to see this surgeon in the beginning, and then they're telling you you've got to wait for another three months to quit smoking… And sometimes it just feels like enough is enough.”

Pharmacists approved of the requirement but acknowledged they did not have all of the evidence regarding possible complications associated with smoking.

Some PCPs felt a more thorough explanation of smoking-related complications would highlight the importance of quitting prior to surgery and would result in increased quit attempts. Like one PCP (35 (VA)) commented, “I'd say the vast majority [of patients] don't [understand the importance of quitting before surgery]. And so when I tell them why, then it seems to make more sense.”

### 3.4. Patients

Almost all patients expressed strong opinions either for or against the requirement. About half of the patient participants reported that they felt the requirement was beneficial and could not only help people get motivated to quit but would also improve their overall health, making statements like, “I think [the requirement is] good. I really do. Because us old farts need to quit smoking and get better health” (6P). The remainder thought it should not be a requirement and/or was discriminatory. One patient (1P) said, “If I choose, at the age of [50+], to smoke a damn cigarette, that should be my right to smoke a cigarette.” Another said, “And to sit there and tell [a Veteran], we're not gonna give you medical treatment because you smoke, the guy risked his entire, the person risked their life to serve for the country and… Everybody sits and says, talks about discrimination and everything, but you're gonna discriminate against a person because he smokes, so you're gonna remove his medical care? No, that's wrong” (5P).

(3) Implementation and possible implications of the requirement

### 3.5. Clinicians

PCPs and surgery staff differed regarding their attitudes toward and responses to the strictness of the requirement. Almost all surgery staff described the importance of a straightforward approach. However, one surgeon (3 (VA)) went on to say, “You need to leave some discretion up to the surgeon and the patient, and their discussion to really do the best thing for the patient rather than trying to mandate yet another thing like that.” Surgery staff overall reported being clear about their individual guidelines and acting within them with little exception in order to minimize surgical complications. A few surgery staff clarified though that it is important to remember the somewhat subjective nature of the term “elective” (i.e., patients may not think it is elective) and that when surgery is considered elective, there may be less of a trade-off in conversation between the risks of smoking and the risk of delay.

Most PCPs reported that there should be exceptions for allowing surgery. For instance, one PCP (7 (non-VA) said, “Sometimes I do want to get on the phone and call the surgeon to ask for an exception… I think if someone has exhausted all the options [for quitting] they would still deserve the procedure.” Some felt smoking was so addictive and may feed into a pain/smoking cycle, making it even more difficult for patients to quit. They also expressed a tension with wanting to follow requirements while also staying patient-centered: “I like to give patients choices. And… I'm not exactly giving them a choice if I say, well, you need to quit smoking before you have this surgery. And in reality there are alternatives, and so they could potentially go somewhere else” (8 (VA)). A VA pharmacist (34) said that while they support the requirement generally, “the other part of me is torn in that a lot of patients will never quit smoking. So that just means that not only will they not quit smoking, they also won't get this other surgery that might improve their quality of life.” Many PCPs and pharmacists went on to say there should be exceptions for patients who have tried to quit, who have cut down, or for whom the benefits outweigh the risks. One PCP (38 (VA)) expressed how they would figure out how to get an exception by referring patients “to the community [i.e., VA-purchased care]. Just have a workaround.” Pharmacists, like some PCPs, were unsure if it would result in long-term abstinence. One non-VA PCP (31) noted, “I've seen a lot of people quit smoking for a procedure and then they get it done and then a month later they just start back up… They're doing for a means to an end.” Correspondingly, one PCP said they would tell patients explicitly that they could start smoking again after surgery.

Clinicians overall expressed little concern that the requirement might increase rural-urban disparities, although some noted the culture of smoking in rural areas could make quitting more difficult. One VA PCP (17) noted what many echoed that smoking is a “cultural habit. And that's hard to address from a distance. And giving them an app on their phone when they have a Nokia flip phone from 1982 is not gonna support that. For many people smoking is cultural, it's social, it's what their spouse does, what they do with their families. Especially in rural areas where smoking is more accepted. So to expect a rural Veteran, even if I mail ‘em some gum, or some lozenges or some patches, to be able to tackle the social component and the habit component of smoking on their own is tough.”

Most concerns were over transportation, internet/phone service issues, and tech savviness regarding an intervention if it included telehealth; however, most clinicians commended VA's ability to help veterans' access to cessation resources.

### 3.6. Patients

Two patients thought there definitely needed to be exceptions, with one saying, “If what the surgery would solve would make [patients] healthier, then it can overshadow the fact they smoke… I would hope that if the surgery really needed to be done, [clinicians] would overlook that” (2P). In addition, some patients commented on their other health characteristics influencing their ability and desire to quit smoking for a surgery, especially when smoking is perceived to combat pain and associated stress. A few patients discussed going back to smoking after surgery, so they only had to quit for “30 or 60 days” (6P), or saying, “After [date of surgery], if I go back to smoking, I will go back to smoking. It's my choice. It's not their choice it's my choice… I've done their part” (9P). A couple of patients also mentioned asking for referrals to community surgeons who did not require patients to quit smoking preoperatively.

Similarly to clinicians, a few patients discussed the culture of smoking in rural areas as a barrier to quitting. Many noted the benefits of the VA pharmacy in mailing cessation resources and appreciated their clinicians' frequent offers to help them quit. Again, there were concerns about transportation and internet/phone issues if resources required face-to-face visits and/or telehealth, respectively. They suggested clinicians offer all cessation options and modes to patients.

## 4. Discussion

Orthopaedic surgery staff and some PCPs were aware of the requirement for patients to quit smoking prior to elective orthopaedic surgery. Neither VA nor non-VA PCPs, and few patients, knew the specifics of the requirement. While PCPs were generally aware of the existence of this requirement, they, along with most patients, were unclear of the details, such as the duration of preoperative abstinence or the need for biochemical verification. These results suggest a missed opportunity to deliver evidence-based pharmacological and behavioral interventions for treating tobacco use and dependence [18]. Given the importance of educating patients on both the surgery-specific benefits of quitting and the process that needs to be followed to adhere to the cessation requirement, clinicians of all specialties involved should be provided training and/or resources to ensure they discuss the pertinent details with their patients. Indeed, staff training has been shown to be helpful in encouraging smoking cessation [15, 16]. There may also need to be more interservice communications regarding the requirement details and the evidence supporting the preoperative smoking cessation requirement.

A nationwide survey found that when there were higher clinician-reported levels of communication between primary care and specialist physicians, there were lower rates of potentially avoidable hospitalizations, especially when using health information technology tools like electronic health records or care navigators [19]. Within and outside VA though, primary care may find challenges communicating via the EHR, especially because rejection of orthopaedic referrals on account of not meeting certain eligibility criteria, such as the requirement for preoperative abstinence, is often a closed-loop interaction. Communication via the EHR is also particularly difficult as non-VA and VA providers are not on the same EHR platform. PCPs may need to resort to telephone or email contact; however, face-to-face contact is likely the most beneficial in discussing specialty care referrals [20].

Almost all patients expressed a lack of knowledge about the reasons behind the requirement, which could influence attitudes and behaviors toward smoking behaviors before and after surgery. Certainly, awareness of the benefits of quitting can lead to a “teachable moment,” which is an event that occurs naturally in the life course that is “thought to motivate individuals to spontaneously adopt risk-reducing health behaviors.” [21] Teachable moments may be assisted by high-quality communication from clinicians. Providing information on the consequences of smoking perioperatively, especially in a face-to-face format, along with reviewing goals and giving options for additional longitudinal support postprovision of information, may be helpful prior to surgeries [22–24]. It is unknown whether the intentions of some patients in our study to return to smoking after their surgery would have been the same if they had been well-informed and connected to cessation treatment resources preoperatively.

In one study, patients undergoing thoracic surgeries were nearly twice as likely to quit smoking compared with those who did not undergo surgery, especially among those who used a Quitline or other intervention [25]. While those patients were undergoing thoracic surgery for cancer (i.e., nonelective), there may be similar motivations to quit in our sample in that surgical complications would remain a facilitator of cessation [26]. Participants in our study revealed mixed opinions as to whether or not there should be exceptions, despite some agreeing it could be a motivator, potentially due to the lack of knowledge about smoking-related surgical complications as noted above. Since surgery staff are more likely to be familiar with literature surrounding pre- and postoperative orthopaedic health, showing that the longer a person has quit smoking prior to surgery translates into decreased incidence of postoperative complications, it is not surprising that they expressed stronger opinions about not allowing exceptions [27, 28].

Next, we are developing the following intervention, considering the potential cultural and technological barriers for rural-residing patients. Among patients who smoke and have an orthopaedic surgery consult ordered, PCPs will provide brief advice and an educational handout our study team and collaborators created on the benefits of quitting smoking perioperatively. Next, they will refer patients to the pharmacist who will contact the patient to provide longitudinal cessation assistance either in-person or via telehealth. We will test the intervention to determine behaviors related to the requirement and intervention, such as if patients quit smoking more successfully. We will also evaluate implementation barriers and facilitators.

Our study does have limitations. Our sample was comprised of an older, white patient population from two VISNs that is not representative of all Veteran or clinician experiences. This study may suffer from selection bias, moderator acceptance, and recall bias. The timing of data collection may not have allowed us to capture all feelings and attitudes because each interview reflects one snapshot in time. We only assessed the knowledge and attitudes of clinicians and patients, not the causal impact of the requirement on behavior change. Finally, we did not specifically ask about requirements regarding cessation of other tobacco products, including e-cigarettes, or the rationale behind some surgeons not allowing preoperative use of nicotine replacement products (NRT). Although data are limited, there is no evidence of NRT increasing surgery complications. Continued research is needed to inform recommendations related to the use of other tobacco and nicotine products during the perioperative period [29, 30].

## 5. Conclusion

Most participants thought elective surgery was a good motivator to quit smoking; but PCPs, surgery staff, and patients differed on whether there should be exceptions to the requirement that patients quit preoperatively. Patients and some PCPs requested more information about the reasons behind the requirement. Future efforts to augment perioperative smoking cessation may benefit from educating patients more about the surgery-related benefits of quitting across primary care and surgical services.

## Figures and Tables

**Table 1:** Self-described participant characteristics.

Patient characteristic (*N* = 26)	*N* (%) or mean (SD)
Age (yr.)	61 (8.8)
Gender	
Male	24 (92%)
Female	2 (8%)
Race	
White/Caucasian	24 (92%)
More than one race	2 (8%)
Ethnicity	
Hispanic or Latino	1 (4%)
Not Hispanic or Latino	25 (96%)
Highest education completed	
High school	8 (31%)
Some college	12 (46%)
College	4 (15%)
Graduate school	2 (8%)
Employment	
Full-time	2 (8%)
Part-time	1 (4%)
Self-employed	1 (4%)
Retired	16 (62%)
Disabled	6 (23%)
Annual income	$44,000 (27,000)
Marital status	
Married	11 (42%)
Divorced	12 (46%)
Never married	3 (12%)
Smoking status	
Current	11 (42%)
Cigarettes per day for current smokers	11 (7.3)
Former (no cigarettes within the past 30 days)	15 (58%)
History of e-cigarette use	
Yes	4 (15%)
No	22 (85%)
Pack years	35 (21)
Surgery	
Yes	10 (38%)
No	16 (62%)

Clinician characteristic (*N* = 38)	N (%)^∗^ or Mean (SD)
Age (yr.)	46 (12)
Gender	
Male	20 (53%)
Female	18 (47%)
Race	
White/Caucasian	27 (71%)
Asian	5 (13%)
More than one race	3 (8%)
Other	1 (3%)
Missing response	2 (5%)
Ethnicity	
Hispanic or Latino	0 (0%)
Not Hispanic or Latino	36 (95%)
Missing response	2 (5%)
Smoking status	
Former	6 (16%)
Never	30 (79%)
Missing response	2 (5%)
Specialty	
Surgery	10 (26%)
Primary care	23 (61%)
Pharmacy	4 (11%)
Other	1 (3%)
Role	
Attending physician	20 (53%)
Resident physician	1 (3%)
Advanced practice provider	13 (34%)
Nurse (RN)	1 (3%)
Other clinic staff	2 (5%)
Student	1 (3%)
Years in practice	14 (10)
VA/non-VA	
VA	28 (74%)
Non-VA	10 (26%)
Years at site	6.9 (6.3)

## Data Availability

Participants did not consent to their data being shared.

## Appendix

### A. Interview Guides

#### A.1. Clinicians

Smoking Cessation and Elective Surgery Among Rural Veterans

We are conducting a study to understand the impact of policies and guidelines related to smoking cessation among Veterans prior to elective surgeries. We want to talk to Veterans and clinicians about their experiences and thoughts around this topic.

(1) First, please tell me about yourself (role, background, etc.)
(2) Next, please tell me about your health care institution (location, rural/urban, patient makeup, etc.)
  - Of the patients you saw in the past year, can you provide an estimate what percent were rural Veterans?
  - In the past year, what is your best estimate of the percent of your patients who reported current smoking at the time of request for an elective surgery?
(3) In general, what is your role in treating tobacco?
  (c) Are there certain places or people that you refer your patients who want to quit smoking?

Now, I would like to know about your awareness and experiences related to policies or standards of care that require patients to stop smoking prior to elective surgery.

(4) Are you aware of any such policies or standards of care? What is your understanding of this policy/standard of care?
  - Are you aware of any specific to the VA? [*If no, ask the remainder of the questions about the policy they are aware of]*

We are interested in your experiences with the policy or standard of care that is specific to the VA [*If not familiar with the VA policy/standard of care, ask the remainder of the questions about the policy they are aware of]*

(5) How were you informed about this policy or standard of care?
  - Was there training / support/ other resources around how to support this process?
  - Is there a specific protocol available to you and your colleagues around this policy or standard of care related to quitting smoking prior to surgery?
(6) What are your thoughts around requiring smoking cessation prior to elective surgery?
  (c) What impact do you think this requirement/guideline of smoking cessation has had on patients access to surgery? On their desire to quit smoking?
  (d) What do you see as potential benefits and concerns of requiring cessation prior to elective surgery?
  (e) [*For surgeons only*]:
    - How often have you needed to delay surgery until a person stopped smoking?
    - Have you made exceptions and allowed a patient who is still smoking to have surgery? What were the circumstances?
    - What happens if you allow a patient to have an elective surgery without quitting smoking? Is this enforced in any way?
(7) Tell me about how you address tobacco use with patients seeking elective surgery. [*For non-VA providers*]: Does this differ for your patients who are Veterans?
  - How do you talk to patients about needing to quit smoking before having surgery?
  - How do patients typically respond?
  - Do you think they understand the relationship between smoking and adverse surgical outcomes?
  - In your experience, are there certain Veterans that are more likely to try to quit smoking before surgery? More likely to be successful? Why do you think that is?
(8) We are particularly interested in increasing access to assistance in quitting among rural Veterans. How can we help these patients to quit?
  - Are there barriers that rural Veterans are more likely to experience than other populations?
  - What are your experiences / thoughts on the use of telehealth among rural Veterans?
  - What would make it easier for you to provide these services to rural Veterans?
(9) Please walk us through the process from when you are first aware a patient who smokes wants surgery to the surgery (or not).
  - How is care coordinated between a patient's surgical team and their primary care team related to the assessment and treatment of smoking prior to and/or after surgery?
  - Are there others involved in this care? (e.g., anesthesiologists, behavioral health, outside referrals)?
  - Who assists patients who need to quit smoking before surgery and what resources do they have available (lozenges, counseling, patches, Quitline, medications, etc.)?
  - What do you think makes it easy/hard for patients to access cessation resources?

[*For non-VA providers*]: Do you see differences in the resource availability or access among your patients who are Veterans? [*If yes*]: Please tell me more about that.

(e) How do you know if a patient has quit smoking? Who assesses this?
(f) Who follows-up with patients after surgery to help them to stay quit?
(g) What type of assistance is provided to help them stay quit?

(10) What are your preferences for a cessation intervention in terms of method of delivery (e.g. telehealth, paper, in-person, etc.) and content?
  - Who do you think should provide smoking cessation assistance to patients who want surgery? (e.g., primary care team, surgical team, behavioral health, anesthesiologists)
  - Where in the process?
(11) How important is it that patients who want elective surgery are able to get it vs. making sure they quit smoking first?
(12) What other questions should we examine as researchers?

#### A.2. Patients

Smoking Cessation and Elective Surgery Among Rural Veterans

We are conducting a study to understand the impact of the requirement that Veterans need to stop smoking before elective surgeries. We want to talk to Veterans and clinicians about their experiences with this requirement.

(1) First, tell me a little about yourself.
  - Where are you from?
  - Where do you usually go to get health care (VA, other primary care setting)?
  - How long have you been receiving care at ______? How often do you see your primary care doctor?

Thank you for sharing that information. Like I mentioned, we are interested in experiences of Veterans who had a surgery recently or were interested in having surgery. On xx/xx/xxxx, the Portland VA received a request for a consult from your doctor about surgery for xxxx.

(2) Did anyone mention needing to quit smoking prior to having this surgery?
  - Tell me about this conversation. Who was it with? Did you talk about smoking with anyone else involved in your care? [*If yes, ask questions for each person mentioned*]
  - How did they know you were currently smoking?
  - Were you aware that you would need to quit smoking prior to having this conversation?
  - What reasons were provided for needing to quit prior to surgery?
  - How did this conversation make you feel?
(3) How did knowing that you had to quit smoking before surgery impact your smoking patterns or thoughts about smoking? How did it influence your desire or decision to have surgery?
  - Had you been thinking of quitting prior to talking to your doctor about surgery?
(4) Please tell me what you know about the effects that smoking can have on surgery.
  - Where did you get this information (e.g., doctor, surgeon, internet, written materials)?
  - When did you receive this information (e.g., at surgery consult, when discussing with provider, after surgery)?
  - What was the most helpful information you received about quitting smoking before your surgery? Was there anything that was not helpful?
(5) Were you willing to try to quit smoking prior to having surgery?

[*If no, Go to Question 9*]

[*If yes*] Please walk me through how you came to that decision and what happened next with your attempt to quit.

- Who was involved in helping you to quit? What did they do to help?
- How long did you have to be quit before surgery was scheduled?
- How did your doctors know if you quit smoking or if you were still smoking? Who asked you?

(6) What types of resources were offered to help you quit smoking (medications, counseling, patches, lozenges, Quitline etc.)? Did you accept or use any? Why or why not?
  - Who offered these resources?
  - How helpful were each of these resources for helping you quit?
  - How easy/hard was it to access these resources?
  - At what point were these services offered? (e.g., at surgery consult, when discussing with primary care doctor)
  - If offered medications, what information were you provided around the use of medications to quit smoking?
  - Who followed up with you to see how you were doing in your quit attempt? If not, would that have been helpful?
(7) Were you successful in quitting smoking before your surgery? [*If no, go to question 10*]
  - What do you think helped you to succeed? How was this different from other quit attempts?
  - Of all the people you talked to during appointments leading up to your surgery, who do think was most helpful in your quit attempt? Why?
  - Did you end up having surgery?
(8) [*If had surgery*] Have you smoked since your surgery? [*If no*], What helped you to stay quit? [*If yes*], What do you think would have helped you to stay quit?
  - Tell me about any follow-up from any of your doctors or other members of your health care team related to helping you to stay quit after surgery. [*Go to question 10*]
(9) *[Only ask if “no” to question 5 above]* Please walk me through why you were not willing or able to quit smoking before surgery and what happened next.
  - Were any options offered to help you quit at that time or for if you changed your mind? (e.g., cutting back, referral elsewhere, follow-up) Did you use any of them?
  - What other options were offered in place of surgery?
(10) What do you think a surgeon's role should be in asking about tobacco use and in assisting patients to quit? What about the role of a primary care doctor?
  - Who do you think you would be more willing to listen to about the need to quit before having surgery? Why?
(11) How do you feel about the requirement that patients need to quit smoking prior to having surgery?
  - Do you think there should be exceptions to this requirement? (e.g., type of surgery, certain patients)
  - Can you think of ways in which this requirement might be helpful? Not helpful?

Thank you for sharing your thoughts on these topics. I want to switch gears now and ask about access to healthcare services and your thoughts and experiences around telehealth services.

(12) Have you ever needed care for physical or mental health needs that were not available at your local primary care clinic?
  - Were you able to get services somewhere else? How did this come about?
(13) Have you ever used telehealth, such as the phone, your computer or a computer at your local clinic, for a doctor or other healthcare visit? (If no, would you be willing to use telehealth services? What would be barriers to using them? (e.g., no phone/computer access, no internet, etc,)
  - What types of technology did you use? (e.g., phone, app, computer)
  - What did you like about it? What did you not like?
  - What problems did you have using telehealth services?
  - What do you think could make it better?
(14) Tell me your thoughts around using telehealth services to help Veterans quit smoking before surgery or to stay quit after surgery.
  - Is this something you think would be helpful, especially for Veterans in rural areas? Why or why not?
  - Would you use this service if it was offered when trying to quit smoking before surgery? Why or why not?
  - Would you use this service to help you stay quit after having surgery?
  - What type of things do you think would be helpful to include to help patients that need to quit smoking before having surgery? (access to community support groups, online peer support, text reminders, etc.)
  - What type of technology would you be most likely to use for these services? (e.g., phone, computer, tablet)
  - Please tell me whether you would prefer to receive this assistance at an in-person visit or through technology like the phone or the computer? Why do you prefer one over the other?
(15) Other than any assistance in quitting prior to surgery, have you ever used or been offered assistance in quitting smoking at your local VA clinic or other healthcare setting?
  - What were you offered? Did you use any of the resources?
(16) Are there any other things that you think would be helpful for us to know?
